# Machine learning enabled dual to wideband frequency agile $\:{\varvec{A}\varvec{l}}_{2}{\varvec{O}}_{3}\:$ceramic-based dielectric MIMO antenna for 5G new radio applications

**DOI:** 10.1038/s41598-025-93856-y

**Published:** 2025-03-20

**Authors:** Jayant Kumar Rai, Ajay Kumar Dwivedi, Vivek Singh, Pinku Ranjan, Anand Sharma, Ashish Pandey

**Affiliations:** 1 Department of Electrical and Electronics Engineering, ABV Indian Institute of Information Technology and Management, Gwalior, Madhya Pradesh India; 2 Department of Electronics and Communication Engineering, Nagarjuna College of Engineering and Technology, Bengaluru, Karnataka India; 3 Department of Electronics and Communication Engineering, Motilal Nehru National Institute of Technology Allahabad, Prayagraj, Uttar Pradesh India; 4 Department of Data Science & Engineering, Manipal University Jaipur, Jaipur, Rajasthan India

**Keywords:** Dielectric resonator antenna, Frequency reconfigurable, MIMO, PIN diode, 5G new radio, Machine learning, Engineering, Electrical and electronic engineering

## Abstract

This article presents a dual-band to wideband Frequency Agile (FA) rectangular dielectric resonator (DR) based hybrid MIMO antenna for 5G New Radio (NR) application with connected ground. The DR is made of Al_2_O_3_ (ε_r_ = 9.8) ceramic material. The FA is achieved through the PIN Diode switches. When the PIN Diode is in an “ON” state, it provides dual bands due to the excitation of *TE*_*111*_ mode. When the PIN Diode is in an “OFF” state, it provides wideband characteristics due to the excitation of *TE*_*111*_ and *TE*_*211*_ modes in the rectangular DR. The isolation and gain are achieved by 20 dB and 4.3 dBi, respectively. The maximum tuning range is 49.36. The MIMO performance characteristics are achieved within the allowable range. A good agreement is achieved between the simulated and measured results. The suggested MIMO antenna is optimized through the various ML algorithms in which Random Forest (RF) ML algorithms achieved the highest accuracy more than 99% compared to other ML algorithms for S-parameters prediction. Hence, it is suitable for 5G NR applications.

## Introduction

The several advantages of Frequency Reconfigurable (FR) antennas have contributed to their increasing popularity. By employing several techniques, these antennas can automatically transition between frequencies, radiation patterns, and polarisations, which allows them to replace several antennas^1–3^. The rapid expansion of wireless communications systems, higher data transmission rates, and larger channel capacities are becoming increasingly required to support the maximum number of users within a coverage area and provide continuous access to high-definition video streaming. However, fading becomes a significant challenge in a multipath environment due to surrounding objects or obstacles emphasising the significance of this issue^4–6^.

The development of the multiple-input multiple-output (MIMO) system has been caused by its ability to accommodate higher capacities and quicker data rates without consuming more channel capacity. A MIMO antenna system may meet every modern wireless communication system’s needs. MIMO technology increases system capacity and data throughput by using many antennas on the transmitter and receiving sides without consuming more radio spectrum or power. The MIMO system’s requirements include a compact size, high efficiency, and a minimum level of mutual coupling between ports within the operational frequency range^7,8^. One popular type of MIMO antenna that improves wireless communication is the Microstrip Patch Antenna (MPA). This is a result of the MPA’s low cost and small size. However, the MPA has a very low radiation efficiency due to its significant conductor loss. Alternatively, a dielectric resonator antenna (DRA) is a suitable technique that provides a large bandwidth and high radiation efficiency. Other methods that can be employed to excite DRAs include coaxial feed probes, microstrip feed lines, co-planar waveguides, and aperture coupling^9–14^.

**Fig. 1:**
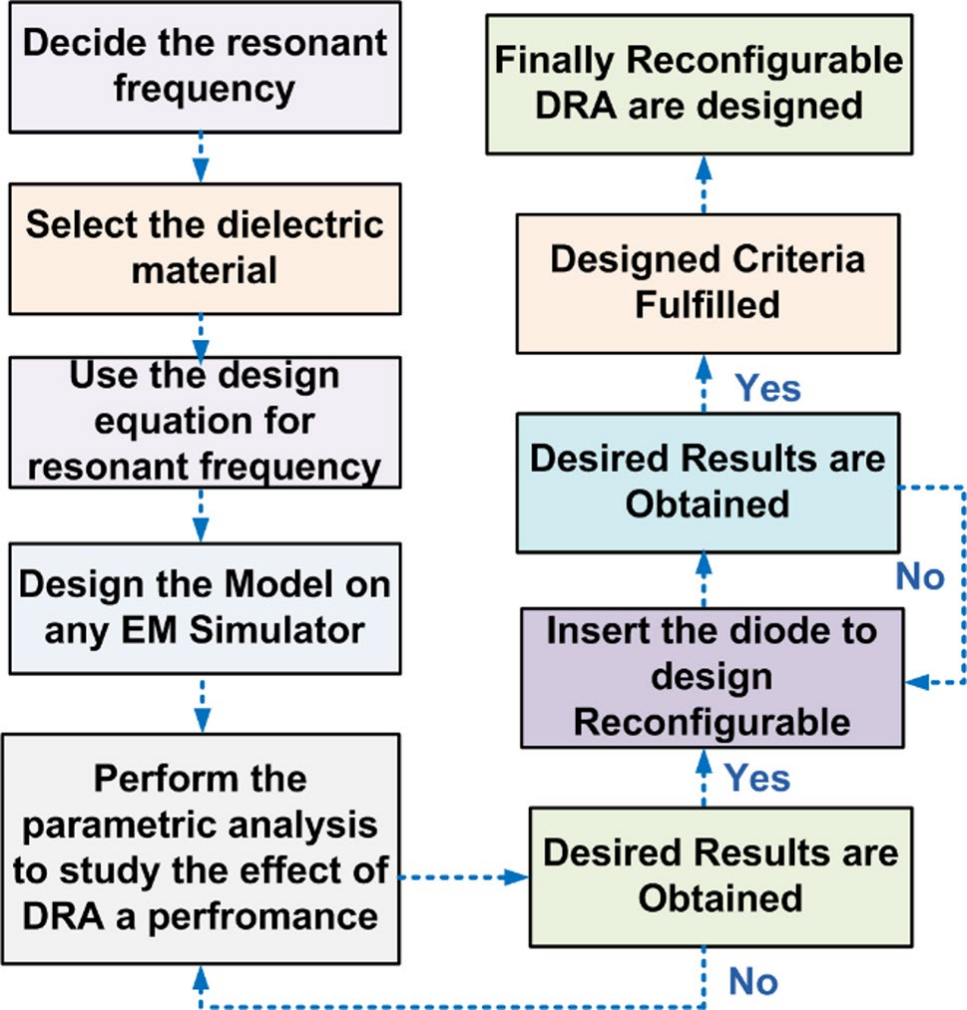
Flowchart for the DRA Based FR MIMO antenna.

Limited research papers regarding DRA-based MIMO dual or triple bands operating in the lower frequency bands are available^4–8,10,11,14^. In^4^, a MIMO DRA allows smartphone multi-band LTE MIMO operation and provides an impedance bandwidth of more than 24% in all frequency bands. In^7^, In all frequency bands, a cylindrical DRA triple-band hybrid MIMO antenna may achieve isolation of more than 25 dB and give an impedance bandwidth of more than 20%. In^8^, cylinder DRA and annular ring produce a two-port dual-band MIMO system. This configuration provides a strong isolation of more than 20 dB for the frequency bands of 1.75 to 2.4 GHz and 3.5 to 5.5 GHz. In^10^, by adjusting the permittivity values, a rectangular MIMO antenna was created that can function in the quad bands of 4.70–4.90 GHz, 3.79–4.04 GHz, 2.83–3.07 GHz, and 5.72–5.86 GHz. In^14^, a dual-band MIMO DRA with permittivity values. The comparative analysis of the suggested FR MIMO antenna with other MIMO antennas is given in Table 1. The flowchart for the DR-based FR antenna is illustrated in Fig. 1.

**Fig. 2:**
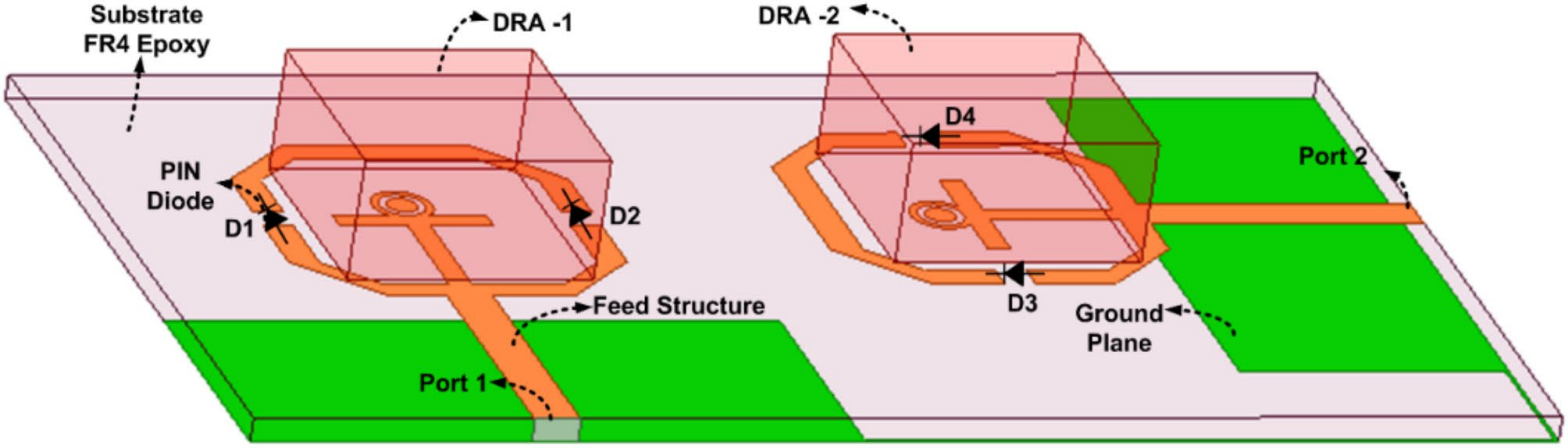
The layout of the suggested FR MIMO antenna.

**Fig. 3:**
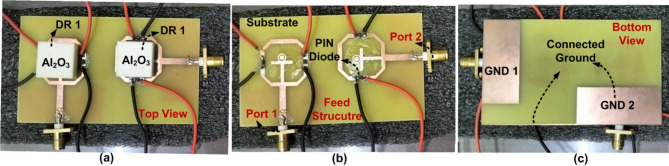
Fabricated prototype.

The layout of the suggested DR-based FR MIMO antenna is illustrated in Fig. 2. and Fig. 3. The attractive characteristics of the suggested DR-based FR MIMO antenna are as follows: a FR MIMO antenna based on DR for 5G Sub 6 GHz applications has been presented. It provides dual-band to wideband characteristics in ON-ON and OFF-OFF configurations, respectively. The isolation and gain are achieved by 20 dB and 4.3 dBi, respectively. The antenna can operate and is adaptable across many frequencies due to its 49.36 tuning range (TR). It is simple in design and easy to fabricate.

**Table 1:** Comparative Analysis of Proposed Work with Other Existing Work.

Ref.	Band (Port)	DR ($\:{\varepsilon}_{r}$)	FR (GHz)	Imp BW (%)	BW (GHz)	ECC	Reconfigurable	Tuning Range	Machine Learning
^4^	Dual (2)	Rectangular (10)	3.40–3.70 5.15–5.35	8.45 3.81	0.3 0.2	< 0.1	No	No	No
^5^	Triple (2)	Cylindrical (9.8)	1.50–2.55 3.21-4.00 4.59–5.98	51.85 21.91 26.30	1.05 0.79 1.39	< 0.16	No	No	No
^7^	Dual (2)	L Shape (10)	3.42–3.80 4.97–5.50	10.52 10.12	0.38 0.53	<0.13	No	No	No
^8^	Dual (4)	Rectangular (37)	0.75–0.96 1.76–3.60	23.97 68.66	0.21 1.84	< 0.5	No	No	No
^10^	Quad (2)	Rectangular (10.2,6.15, 4.3, 2.5)	2.83–3.07 3.79–4.04 4.70–4.90 5.72–5.86	8.14 6.39 4.17 2.42	0.24 0.25 0.2 0.14	Not Given	No	No	No
^11^	Triple (2)	Rectangular (10)	1.63–1.84 2.43–2.71 3.28–3.73	12.10 10.89 12.84	0.21 0.28 0.45	< 0.02	No	No	No
^14^	Dual (2)	Cylindrical (9.8)	1.75–2.40 3.50–5.50	31.32 44.44	0.65 2	< 0.16	No	No	No
**Proposed** **work**	**Dual** **Wideband**	**Rectangular** **(9.8)**	**2.21–2.9** **4.49–5.64** **2.9–5.65**	**0.69** **1.15** **2.75**	**27.06** **22.70** **64.32**	**< 0.05**	**Yes**	**49.36**	**Yes**

*DR-Dielectric Resonator FR-Frequency Range, BW-Bandwidth, Imp BW-Impedance Bandwidth.

### Antenna configuration and analysis

#### Single Port FR antenna

**Fig. 4:**
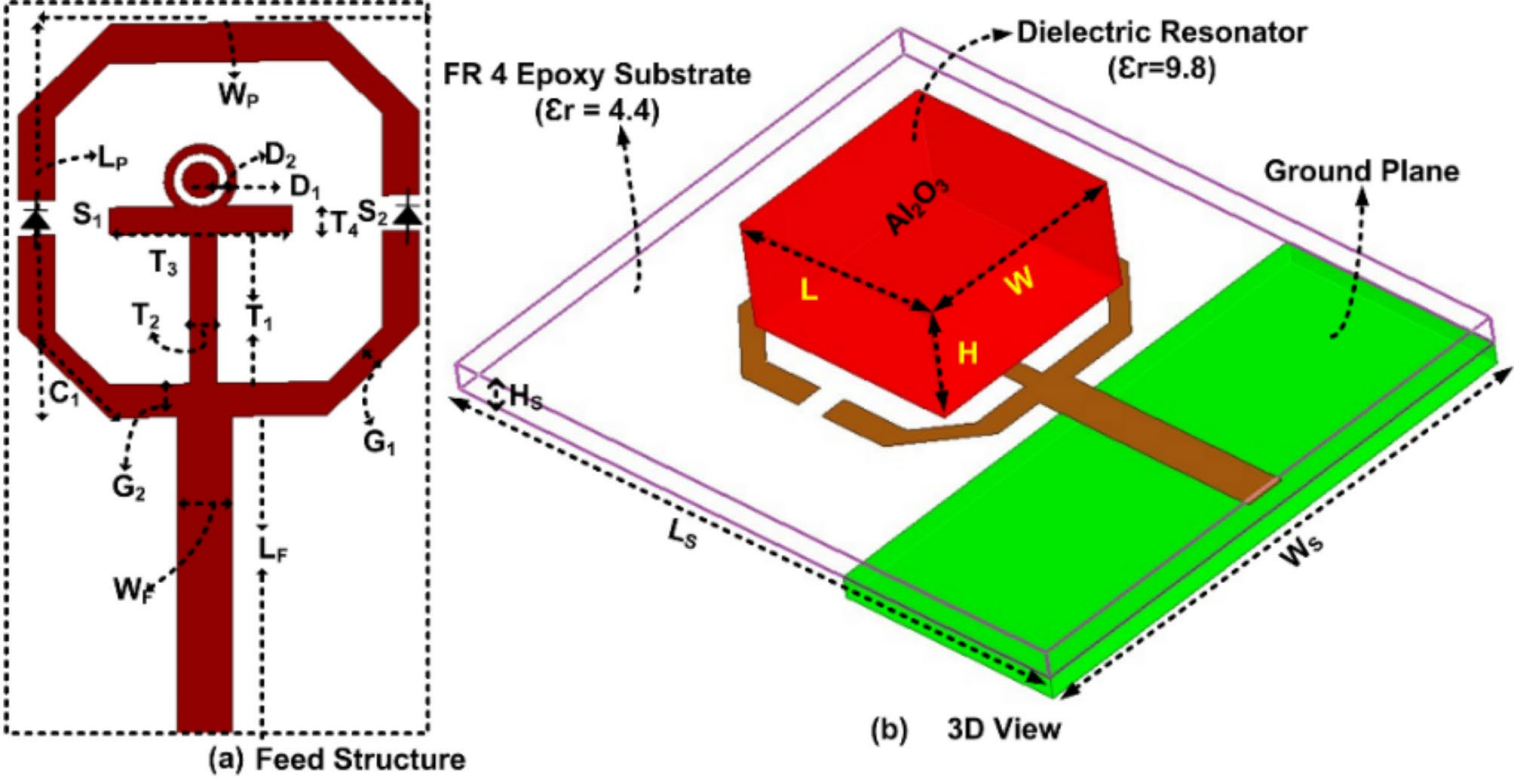
Layout of single port antenna.

The layout of the single port FR antenna is illustrated in Fig. 4. The single port is created on the FR-4 epoxy substrate ($\:{\in\:}_{r}=4.4)\left(tan\delta\:=0.02\right).$ The size of the FR-4 epoxy is 50 × 40 $\:{mm}^{2}$. The ground plane is printed below the substrate. The dimension of the ground plane is 18 × 40 $\:{mm}^{2}$. The dielectric resonator (DR) is made of $\:{Al}_{2}{O}_{3}({\in\:}_{r}=9.8)$ ceramic material. DR is placed above the feed structure. The microstrip patch behaves as a feed for DR. It is created above the substrate. By adding a small quantity of low-loss adhesive material to the dielectric resonator’s contact surface and carefully positioning it in the correct place on the radiator, the RDRs have been securely attached on the radiator’s surface in the suggested configuration. The dimensions of the layout parameters are given in Table 2.

#### Step evaluation of single Port antenna

**Fig. 5:**
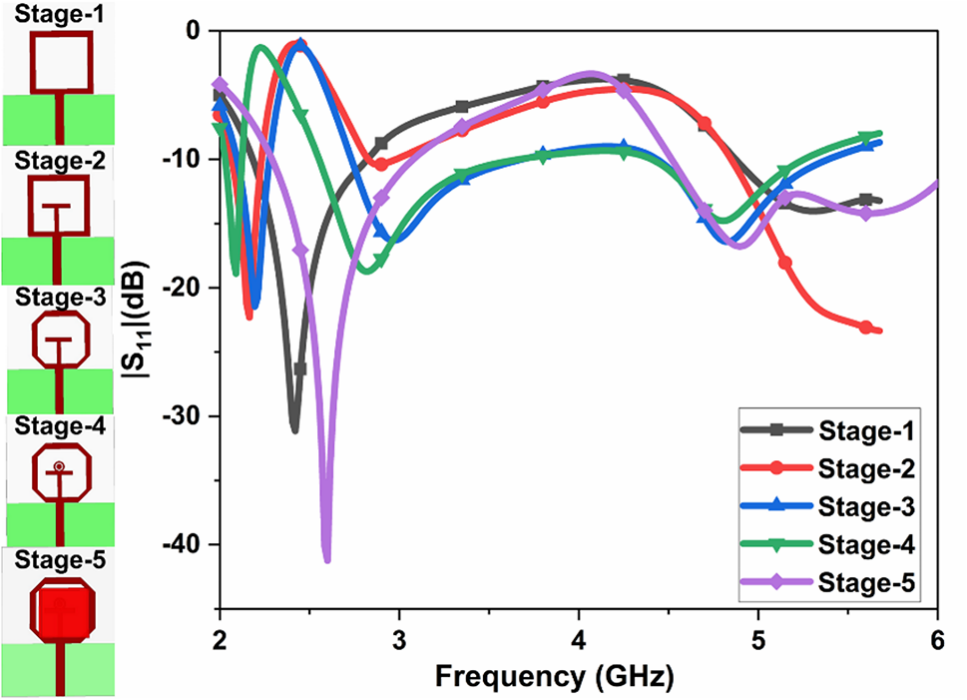
Effect of $\:{|S}_{11}|$ due to various design steps

The effect of $\:{|S}_{11}|$ due to various design steps is illustrated in Fig. 5. In the initial Stage-1, The rectangular patch with half ground is created and resonates at 2.4 GHz. In the next Stage-2, a T-shape is created inside the Stage-1. The resonant frequency is shifted to the left side, and it.

**Table 2:** Dimensions of the layout parameters.

Parameters	Size (mm)	Parameters	Size (mm)
$\:{L}_{G}$	$\:17.5$	$\:{W}_{F}$	$\:3$
$\:{H}_{S}$	$\:1.6$	$\:{\:L}_{F}$	$\:19$
$\:{D}_{1}$	$\:6$	$\:{D}_{2}$	$\:5$
$\:{G}_{2}$	$\:4$	$\:{C}_{1}$	$\:5$
$\:{L}_{C}$	5	$\:{L}_{P}={W}_{P}$	22
$\:{W}_{S}={W}_{G}$	$\:40$	$\:{L}_{S}$	50
$\:{R}_{L}={R}_{W}$	$\:16$	$\:{R}_{H}$	$\:8$
$\:{T}_{1}={T}_{3}$	10	$\:{T}_{2}={T}_{4}$	1.5

resonant at 2.2 GHz. In Stage 3, the rectangular patch’s corner edges are truncated, providing a narrow band and wideband. In Stage 4, the annular ring structure is created above the T-shape, and it also provides the narrowband and wideband, but the resonant frequency is shifted to the left side. In the last Stage-5, DR is placed above Stage-4, providing a narrow band and wideband with improved bandwidth and impedance mapping. The frequency range, bandwidth, percentage of impedance bandwidth, and bandwidth ratio of various stages are given in Table 3.

**Table 3:** Stage by stage parameter analysis.

Stage	Band	Frequency Range (GHz)		Bandwidth (GHz)	% of Impedance Bandwidth	Bandwidth Ratio
		$\:{f}_{L}$	$\:{f}_{H}$			
1	Single	2.19	2.79	0.6	24.09	1.27:1
2	Single	2.06	2.24	0.18	8.37	1.08:1
3	Dual	2.73 4.41	3.62 5.37	0.89 0.96	28.07 19.63	1.32:1 1.21:1
4	Dual	2.55 4.46	3.62 5.19	1.07 0.73	34.74 15.14	1.41:1 1.16:1
5	Dual	2.28 4.52	3.08 6	0.8 1.48	29.85 28.13	1.35:1 1.32:1

#### Mathematical modelling

The single port antenna consists of two dielectric layers: FR4 epoxy and alumina ($\:{Al}_{2}{O}_{3}).$ According to^4^, The effective permittivity $\:{\:(\in\:}_{reff})\:$and effective height $\:{(H}_{eff}$), are given by Eqs. (1)-(2):

1 $$\:{\text{H}}_{eff}=\:\text{H}+{\text{H}}_{\text{S}}$$

2 $$\:{\in\:}_{reff}=\frac{{H}_{eff}}{\frac{H}{{\in\:}_{r\:RDRA}}+\frac{{H}_{S}}{{\in\:}_{rSub}}}$$

In^3^, states that the resonance frequency of the fundamental $\:{{TE}_{111}}^{Z}$ is given by

3 $$\:{k}_{z}\text{tan}\left({k}_{z}\frac{d}{2}\right)=\sqrt{{(\in\:}_{reff-1}){{k}^{2}}_{0}-{{k}^{2}}_{z}}$$

Where, $\:{k}_{x},{k}_{y},$and $\:{k}_{z}$ are wave numbers, they fulfil the subsequent formula.

4 $$\:{{\:k}^{2}}_{x}+{{k}^{2}}_{y}+{{k}^{2}}_{z}={(\in\:}_{reff}){{k}^{2}}_{0}$$

$$\:\text{W}\text{h}\text{e}\text{r}\text{e},\:{k}_{x}=\frac{\pi\:}{b}\:\text{a}\text{n}\text{d}\:{k}_{y}=\frac{\pi\:}{b}$$

According to^1^, Tuning Range (TR) and Total Spectrum (TS) are calculated through the Eqs. (5) and (6):

5 $$\%TR = \frac{2(f_{oh}-f_{ol})} {(f_{0h}+f_{0L})}\: \times \:100$$

Where $$\:{f}_{oh}=highest\:resonance\:frequency$$, and

$$\:{f}_{ol}=lowest\:resonance\:frequency$$

6 $$\:TS=\frac{2({f}_{max}-{f}_{min})}{({f}_{max}+{f}_{min})}$$

Where $$\:{f}_{max}=highest\:\:frequency$$, and

$$\:{f}_{min}=lowest\:frequency$$

#### Parametric analysis

**Fig. 6:**
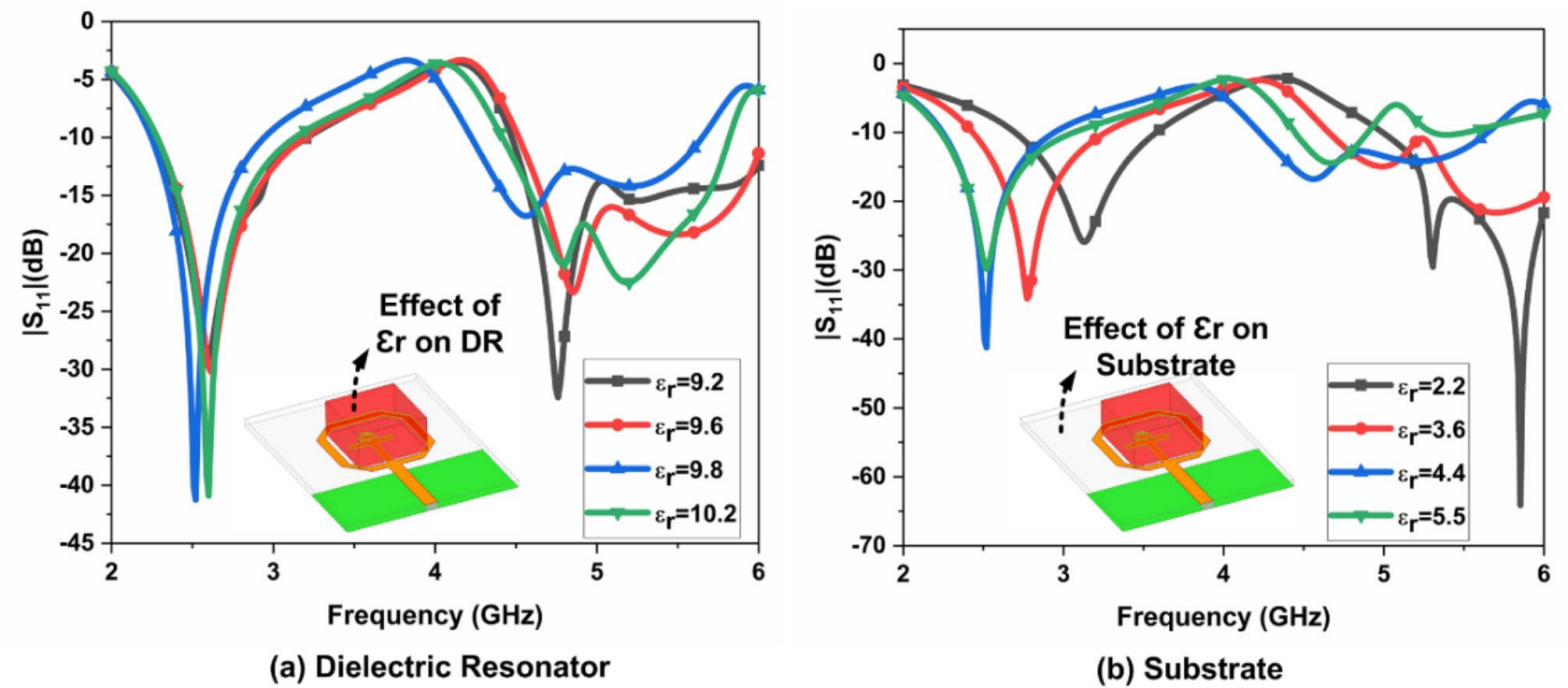
Effect of$\:\:{|S}_{11}|$ due to dielectric constant

**Fig. 7:**
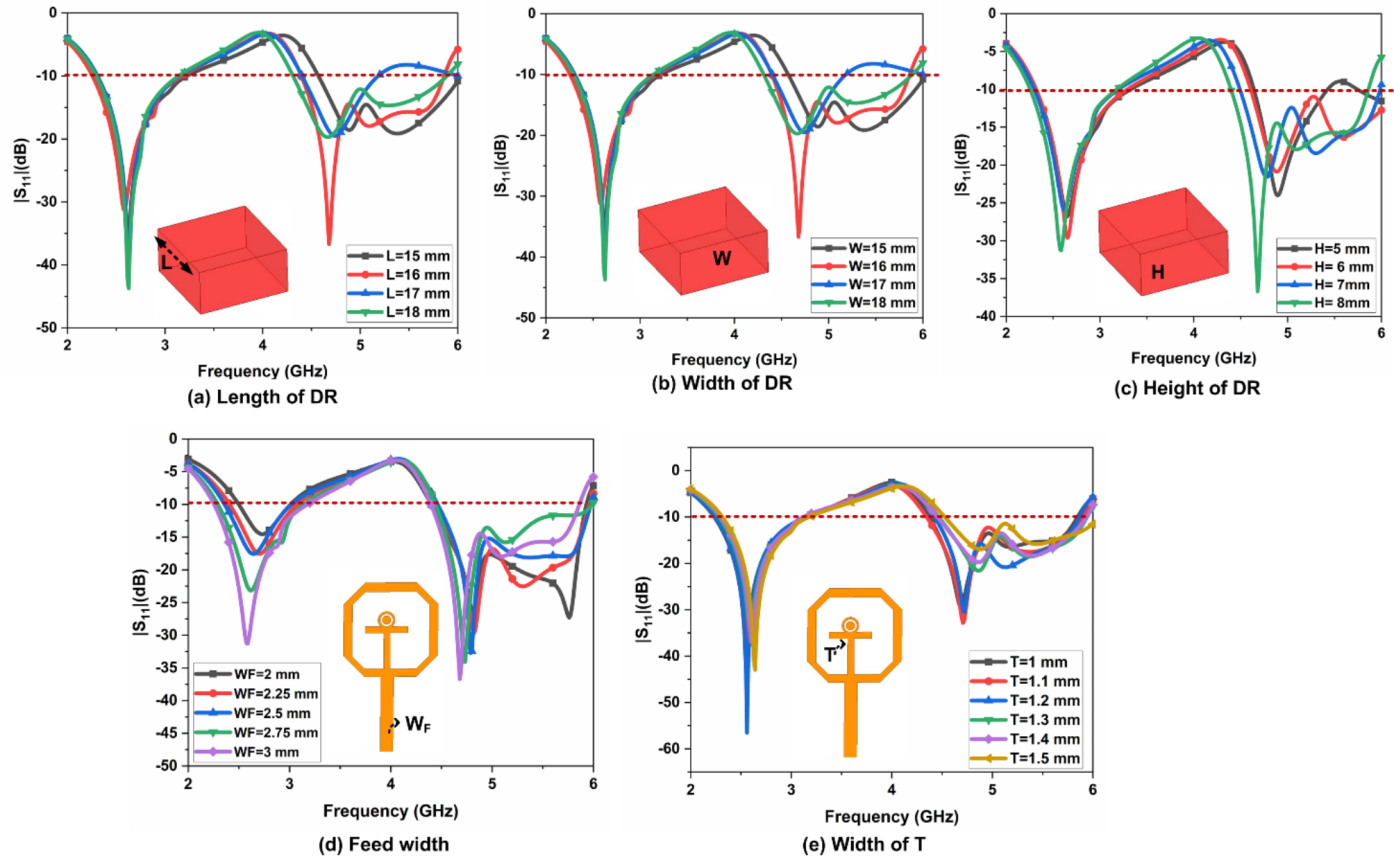
Parametric on various parameters of single port antenna.

The effect of the dielectric constant ($\:{\in\:}_{r})$ on DR and substrate is illustrated in Fig. 6. The dielectric constant of DR varies from 9.2 to 10.2 with a fixed FR-4 epoxy substrate. The $\:{Al}_{2}{O}_{3}$ with FR4 epoxy substrate provides better bandwidth and impedance matching than the others. Similarly, the dielectric constant of the substrate varies from 2.2 to 5.5 with a fixed $\:{Al}_{2}{O}_{3}$ DR. The FR4 epoxy substrate with $\:{Al}_{2}{O}_{3}$ DR provides better bandwidth and impedance matching than the others. The parametric analysis achieves the optimum values of DR’s length, width, and height are illustrated in Fig. 7. The DR’s length, width, and height vary from 15 to 18 mm,15 to 18 mm, and 5 to 8 mm, with a step size of 1 mm, respectively. The L = 16 mm, W = 16 mm, and H = 8 mm achieves better impedance matching and bandwidth than the others. The feed width varies from 2 to 3 mm, with a step size of 0.25 mm. T shape width varies from 1 to 1.5 with a step size of 0.1 mm.

**Fig. 8:**
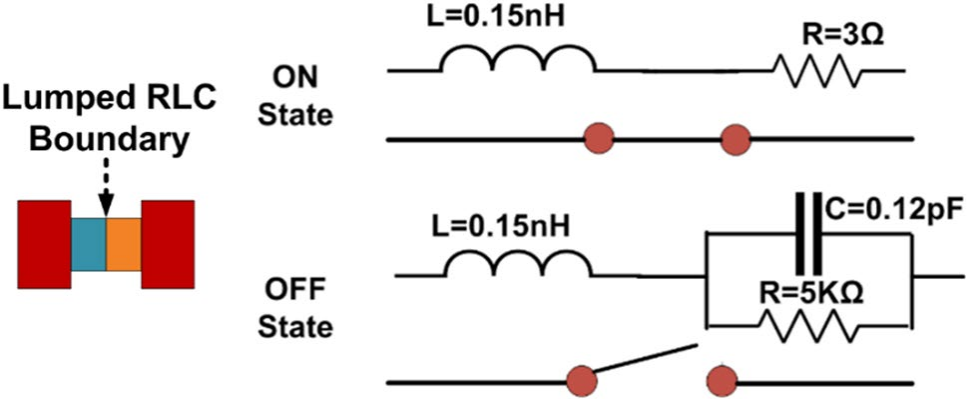
Equivalent Circuit of PIN Diode.

**Fig. 9:**
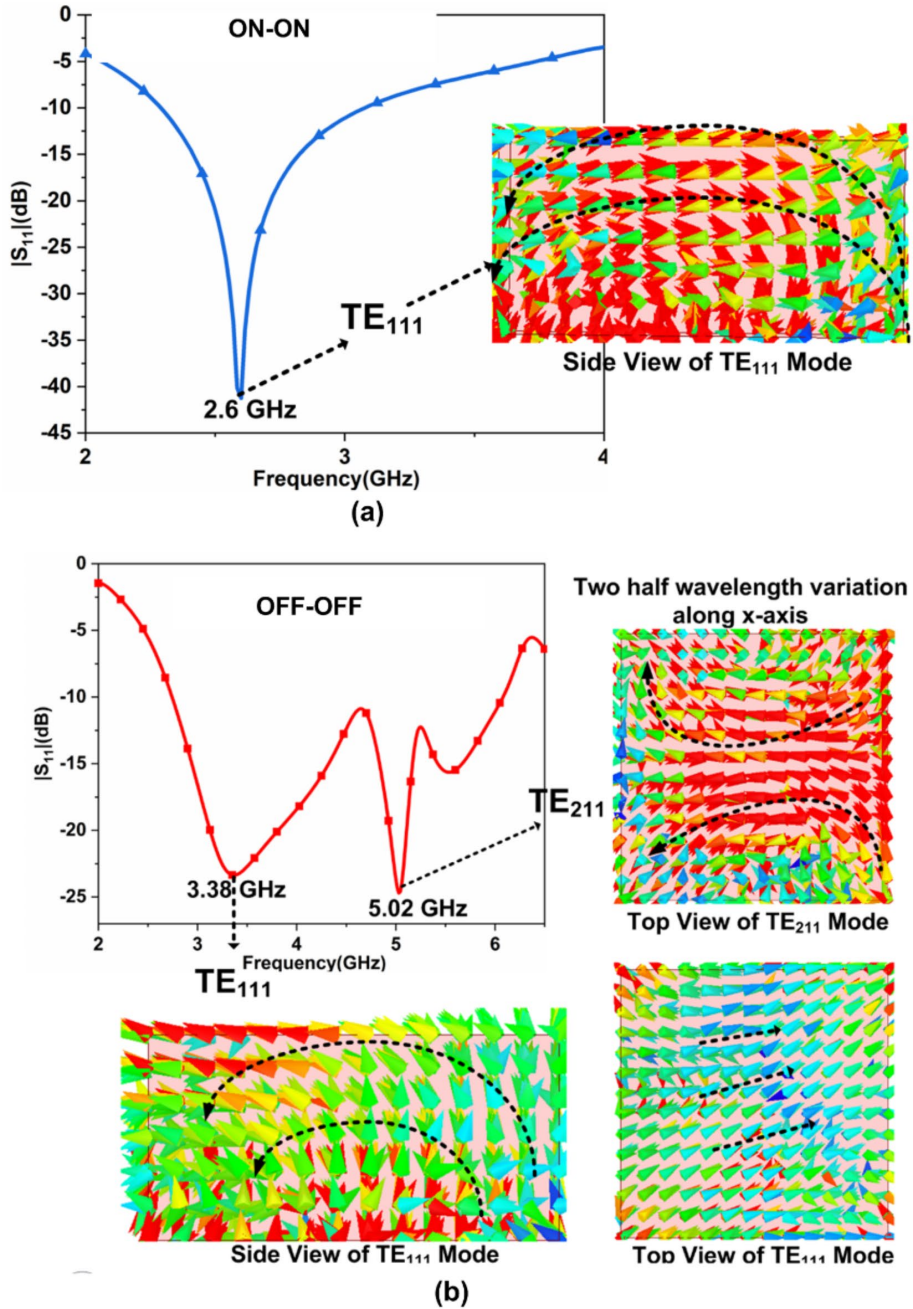
Electric filed distribution in ON-ON and OFF-OFF configuration.

#### Reconfigurable single Port DR

The single-port DR antenna acts as a reconfigurable due to inserting the PIN Diode switches inside the feed structure of DR, as illustrated in Fig. 4. The equivalent circuit of the PIN diode is illustrated in Fig. 8. When the PIN diode is in “ON” state it acts as a series of resistance and inductance. When the PIN diode is “OFF,” it acts as a parallel combination of resistance with capacitance and a series of inductances. PIN diodes have several benefits, including low resistance and lower capacitance values. Additionally, their compact shape minimizes their influence on antenna performance. We used RLC linear components for our simulations even though a PIN diode is a nonlinear element. It should be mentioned that the datasheet is based on actual tests carried out using packaged samples. Even though a diode is a nonlinear device, actual tests are used to get the lumped model. Instead of using a non-linear diode, we can apply a lumped model, and the result will be unchanged^15^. When both the PIN Diode switches are “ON” state, it provides the narrow band. The $\:{TE}_{111}$ mode is generated at 2.6 GHz inside the DR, as illustrated in Fig. 9(a). The wide band is provided when both the PIN Diode switches are in an “OFF” state. The $\:{TE}_{111}$ and $\:{TE}_{211}\:$mode is generated inside the DR at 3.38 GHz and 5.02 GHz, respectively, as illustrated in Fig. 9(b).

#### Two Port DR-based FR MIMO antenna

**Fig. 10:**
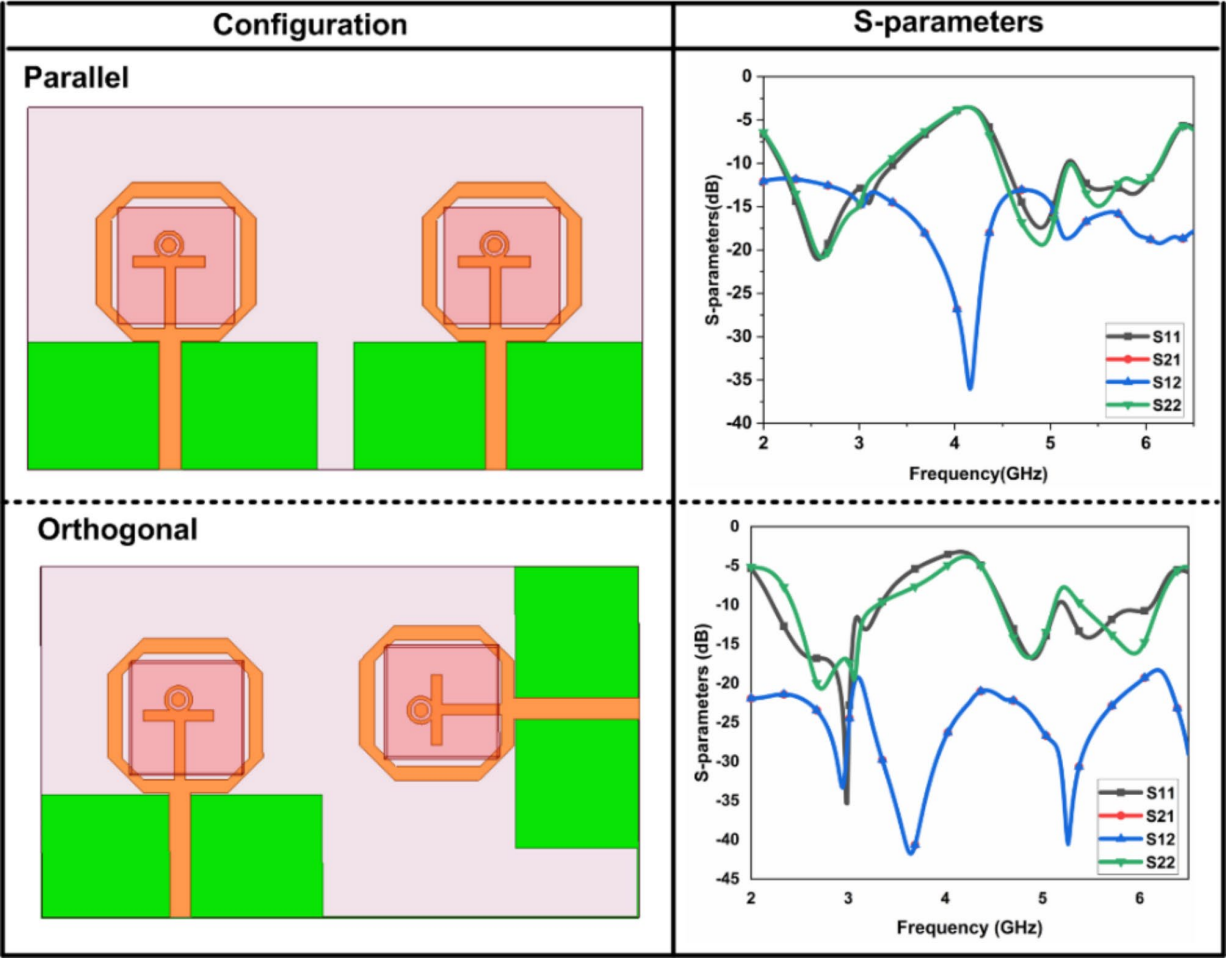
Effect of s-parameters due to configurations.

**Fig. 11:**
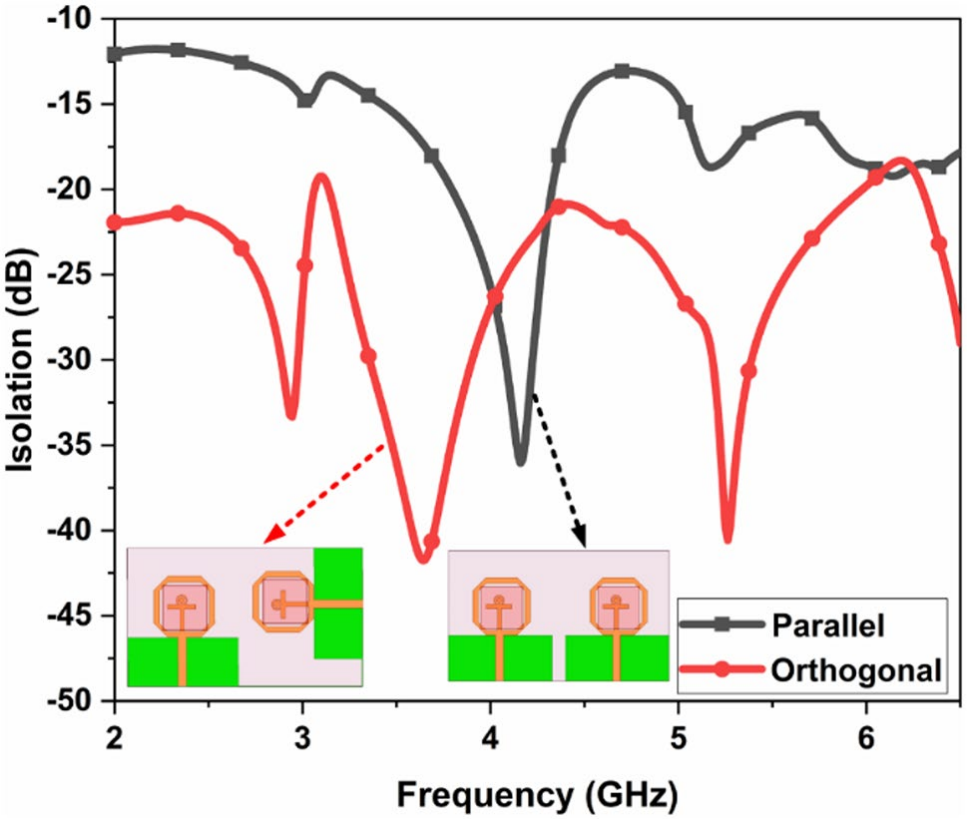
Isolation comparison between the configurations.

The single port DR-based FR antenna is converted into a two port DR-based FR MIMO antenna with an extended substrate. The substrate size is increased to $\:50\:\times\:85\:{mm}^{2}$. The parallel and orthogonal are the two ways to design the MIMO antenna. The comparative analysis between parallel and orthogonal configurations of the proposed antenna is illustrated in Fig. 10. The isolation comparison between the two configurations is illustrated in Fig. 11. The parallel configurations provide an isolation of less than 10 dB, and orthogonal configurations achieve the isolation of 20 dB. To create the Dual port FR antenna, PIN diodes are inserted in the orthogonal configurations, as illustrated in Fig. 2. and Fig. 3. The total numbers of PIN Diodes are increased to four.

## Experimental analysis of MIMO antenna

**Fig. 12:**
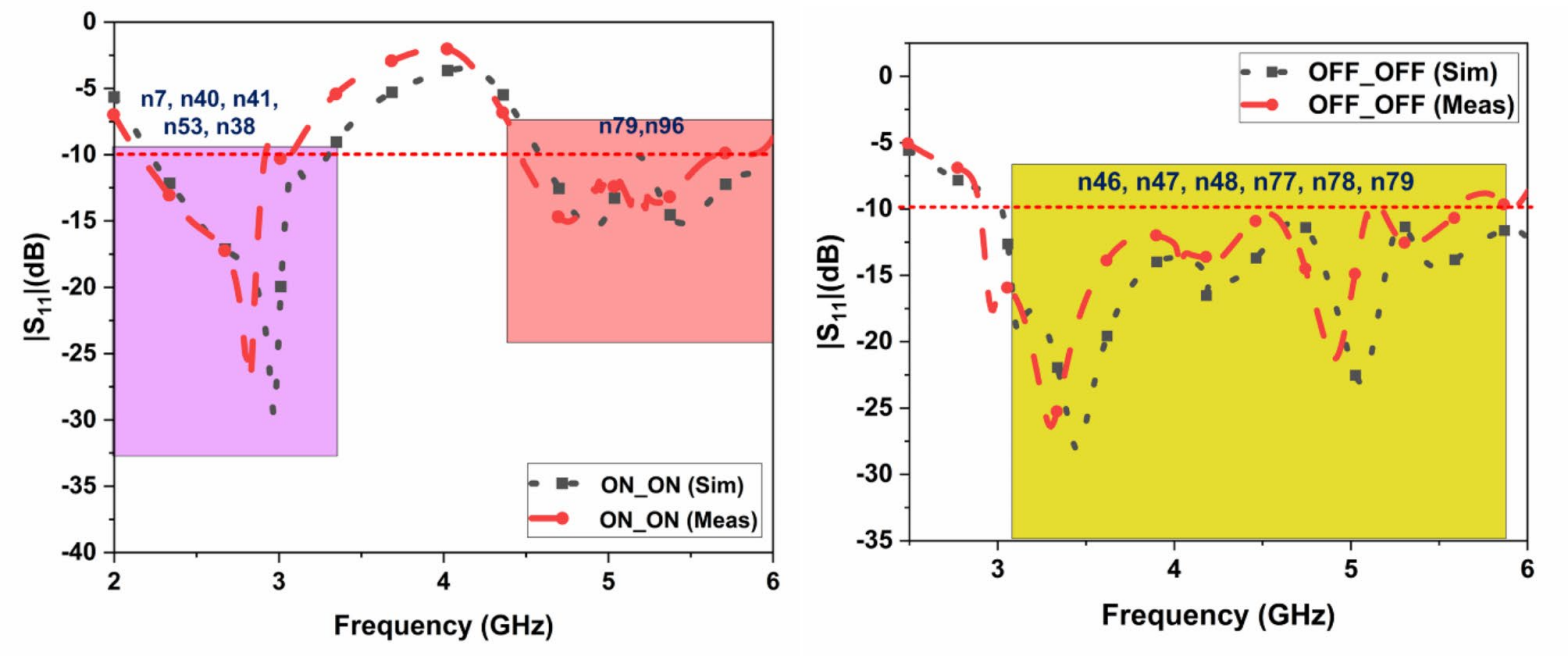
Measured and simulated S_11_ of the FR MIMO antenna.

**Fig. 13:**
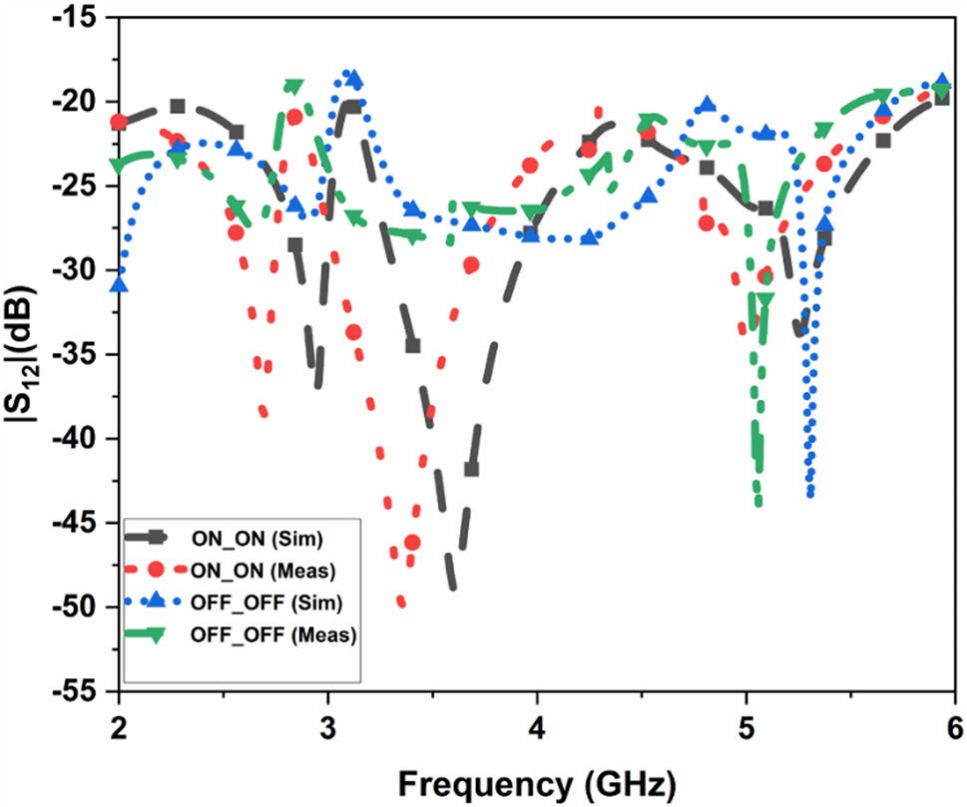
Measured and simulated S_12_ of the FR MIMO antenna.

**Fig. 14:**
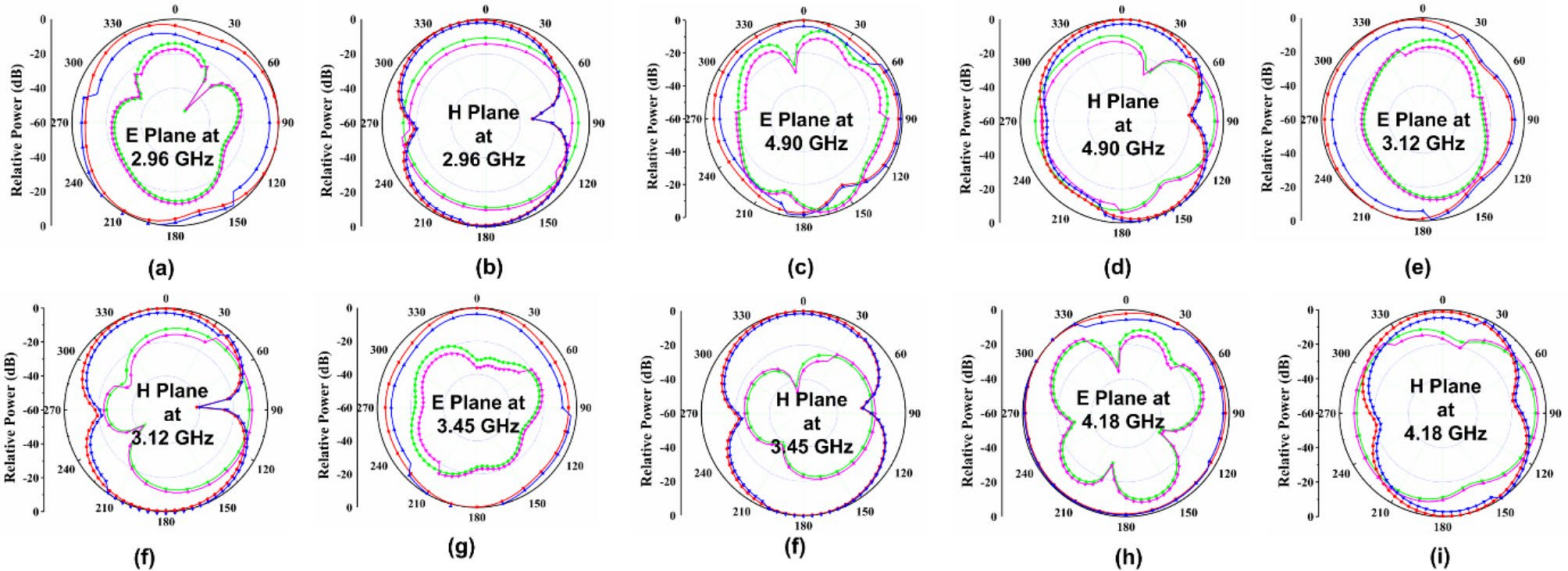
Measured and simulated radiation pattern at E (XZ Plane) and H (YZ Plane) at 2.96, 4.9, 3.12, 3.45, and 4.18 GHz.

**Fig. 15:**
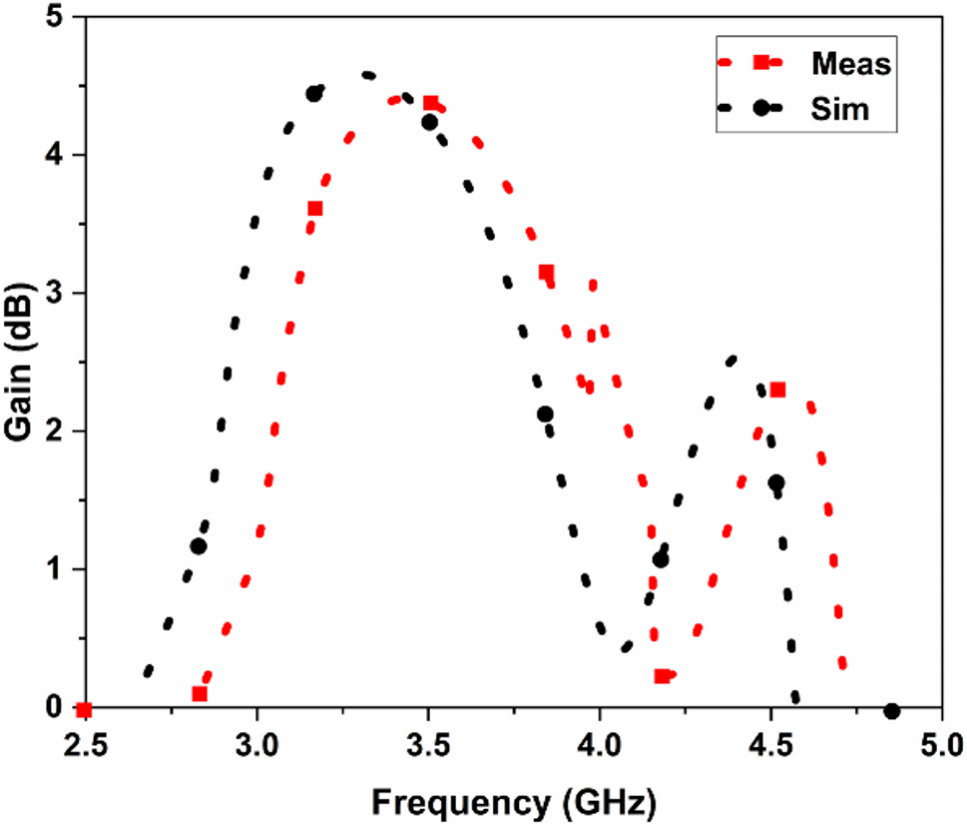
Gain of the proposed of the FR MIMO antenna.

The suggested FR MIMO antenna is fabricated to compare the simulated results, as illustrated in Fig. 3. The measured and simulated |$\:{S}_{11}|$ are illustrated in Fig. 12. The suggested antenna operates in the ON-ON and OFF-OFF configurations. In the ON-ON configurations, the dual-band characteristics are achieved. The frequency ranges are 2.21 to 2.9 and 4.49 to 5.64 GHz with bandwidths of 0.69 and 1.15 GHz, and the percentage of impedance bandwidths are 27.06% and 22.7%, respectively. In the OFF-OFF configurations, the wideband characteristic is achieved at 2.9 to 5.65 GHz, bandwidth of 2.75 GHz, and % of impedance bandwidth is 64.32%. The suggested antenna converts the dual-band into a wideband by switching the PIN Diode to ON and OFF states. The measured and simulated |$\:{S}_{12}|$ are illustrated in Fig. 13. More than 20 dB isolations were achieved in both configurations. The radiation patterns in both E and H planes at various frequencies are illustrated in Fig. 14. the E-panes show omnidirectional, and the H planes show bidirectional radiation patterns at various frequencies. The suggested antenna is a hybrid antenna. The H-plane exhibits a bidirectional pattern due to the magnetic field’s concentration in two directions. Still, the E-plane’s uniform electric field radiates in an omnidirectional direction. The maximum peak gain of 4.3 dBi is achieved and is illustrated in Fig. 15. The performance of the suggested antenna is given in Table 4.

**Table 4:** The performance comparison of the suggested antenna.

Conf.	Band	FR (GHz)		BW (GHz)		% of Imp BW		5G New Radio Bands
		Sim	Meas	Sim	Meas	Sim	Meas	
ON-ON	Dual	2.22–3.29 4.58-6	2.21–2.9 4.49–5.64	1.07 1.42	0.69 1.15	39.6 26.8	27.06 22.70	n7, n38, n40, n41, n53, n79
OFF-OFF	Wide	3–6	2.9–5.65	3	2.75	66.6	64.32	n46, n47, n48, n77, n78, n79

*Conf.- Configuration, FR-Frequency Range, BW-Bandwidth, Imp BW-Impedance Bandwidth, Sim-Simulated, and Meas- Measured.

## MIMO performance parameters

**Fig. 16:**
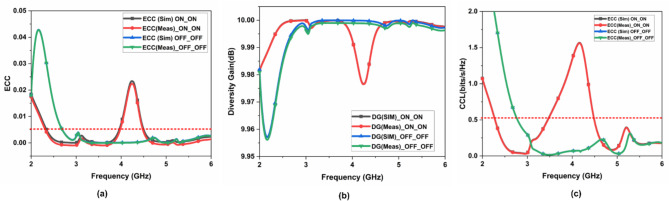
MIMO parameters (a) ECC, (b) DG, and (c) CCL.

**Fig. 17:**
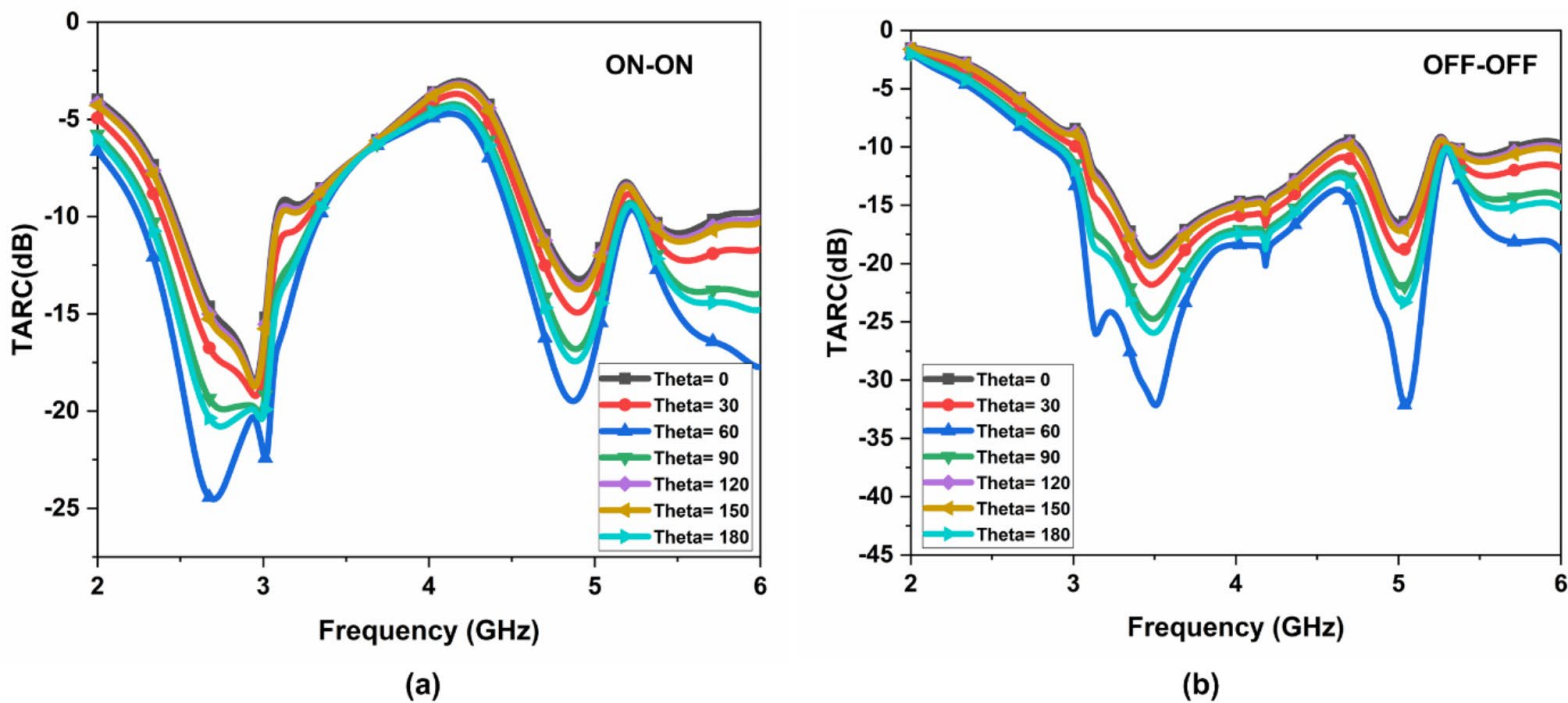
TARC of the suggested FR MIMO Antenna.

The performance of the FR MIMO antenna is determined through the mean effective gain (MEG), diversity gain (DG), envelope correlation coefficient (ECC), and total active reflection coefficient (TARC)^16–19^. The ideal value of ECC is 0.5, DG is around 10 dB, MEG is $\:{}_{-}{}^{+}3$ dB and TARC are < 0 dB. The ECC is determined through Eq. (7), and its value is 0.05. It is illustrated in Fig. 16 (a). The DG is determined through Eq. (8), and its value is 10 dB. It is illustrated in Fig. 16 (b). The CCL value is 0.5 bits/s/Hz. It is illustrated in Fig. 16 (c). The TARC is determined through Eq. (9), and its value is 0 dB. It is illustrated in Fig. 17.

7 $$\:{ECC}_{12}\:=\:\frac{{{{|S}^{*}}_{11\:}{S}_{12}+{{|S}^{*}}_{21\:}{S}_{22}|}^{2}}{(1-{{|S}_{11}|}^{2}-{{|S}_{21}|}^{2})(1-{{|S}_{22}|}^{2}-{{|S}_{12}|}^{2})}$$

8 $$\:DG=10\:\times\:\sqrt{1-{\left|ECC\right|}^{2}}$$

9 $${\rm TARC}\:=\; \:\frac{\sqrt{{(\left|{S}_{11}+{S}_{12}{e}^{j\theta\:}\right|}^{2})+{(\left|{S}_{21}+{S}_{22}{e}^{j\theta\:}\right|}^{2})}}{\sqrt{2}}$$

Where $\:\theta\:$ is input feeding angle

**Fig. 18:**
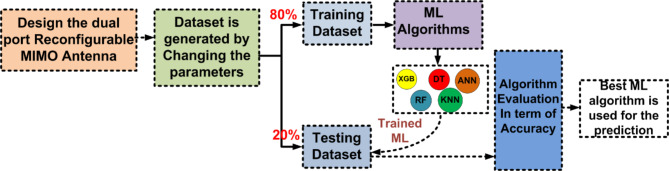
Block diagram of the suggested reconfigurable MIMO antenna.

**Fig. 19:**
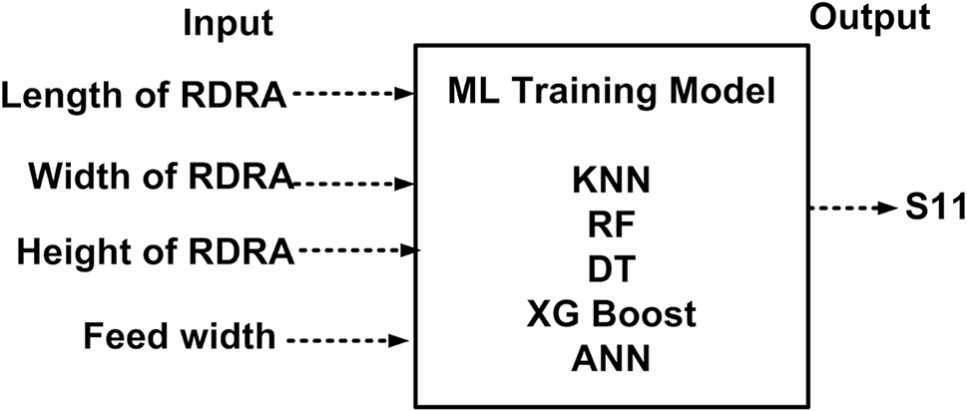
Input parameters for ML models.

## Optimization through machine learning

**Fig. 20:**
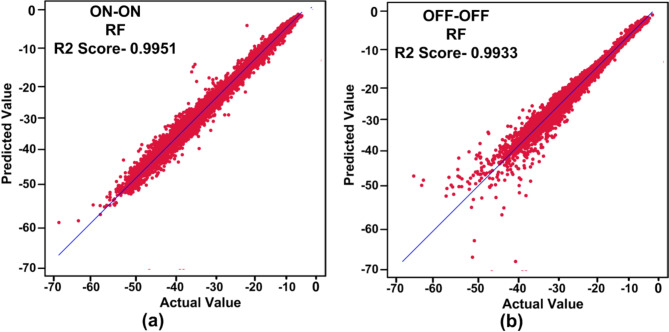
Actual vs. predicted values of RF ML algorithms.

**Fig. 21:**
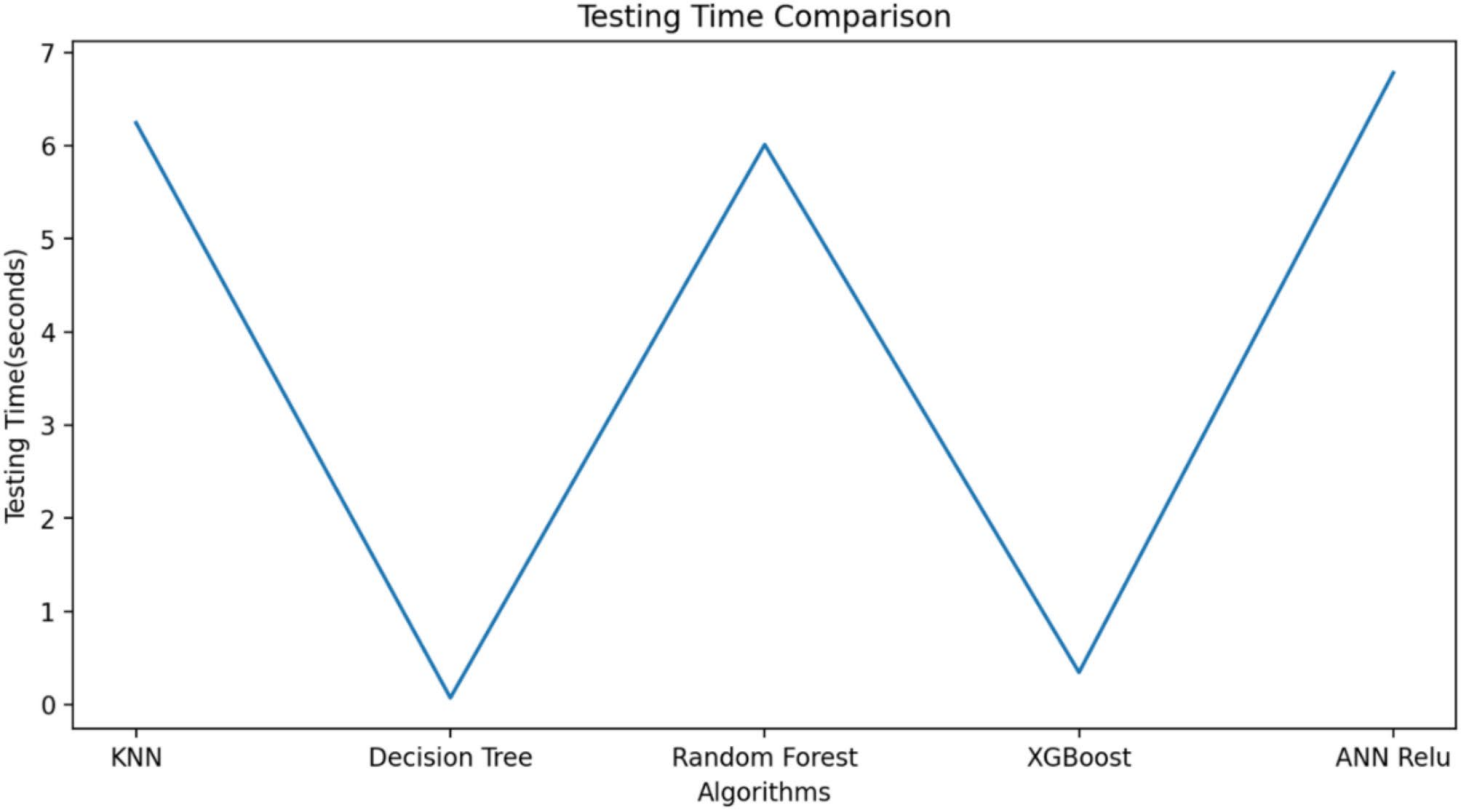
Testing time of various ML algorithms.

**Fig. 22:**
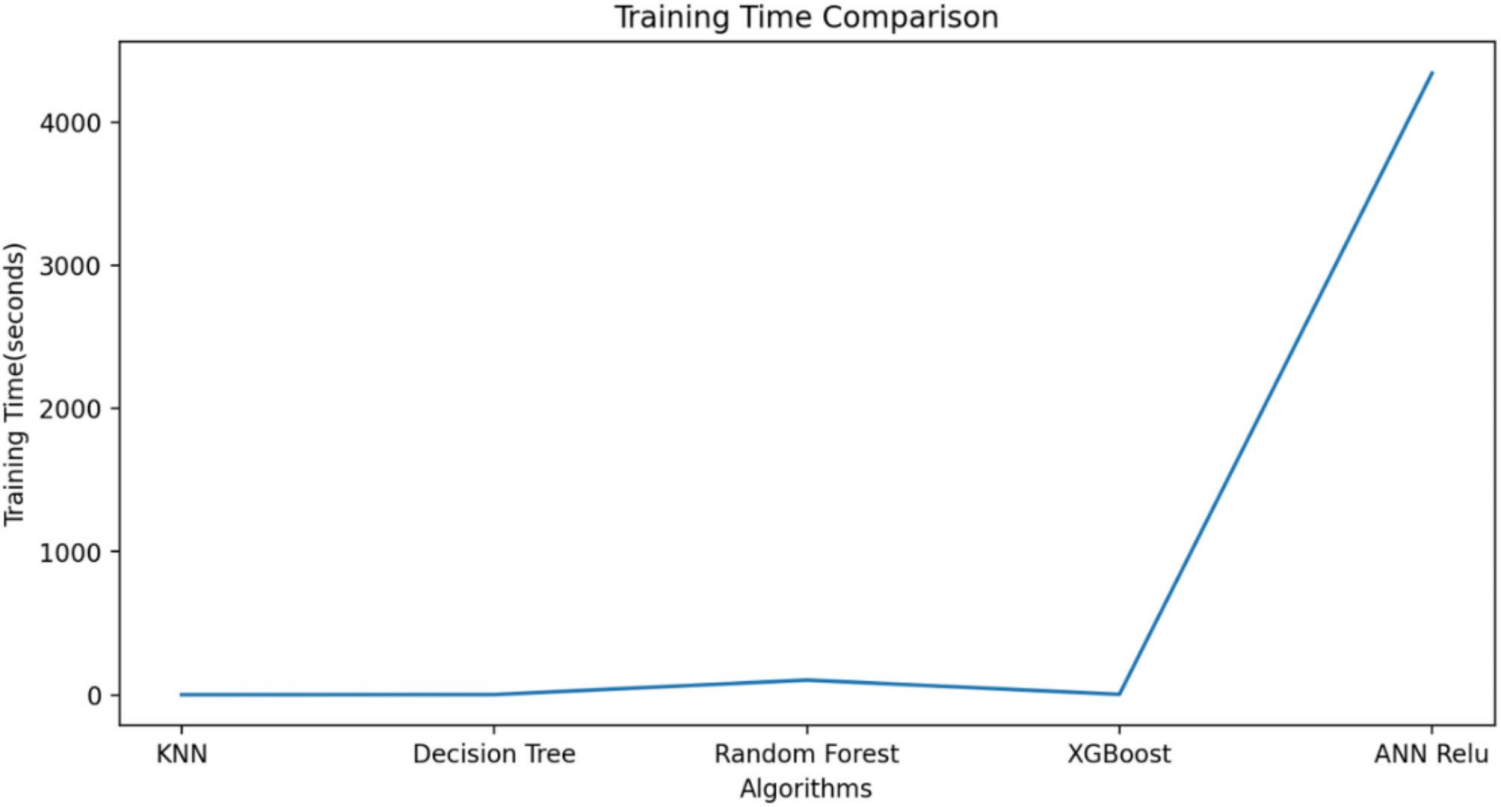
Training time of the various ML algorithms.

**Fig. 23:**
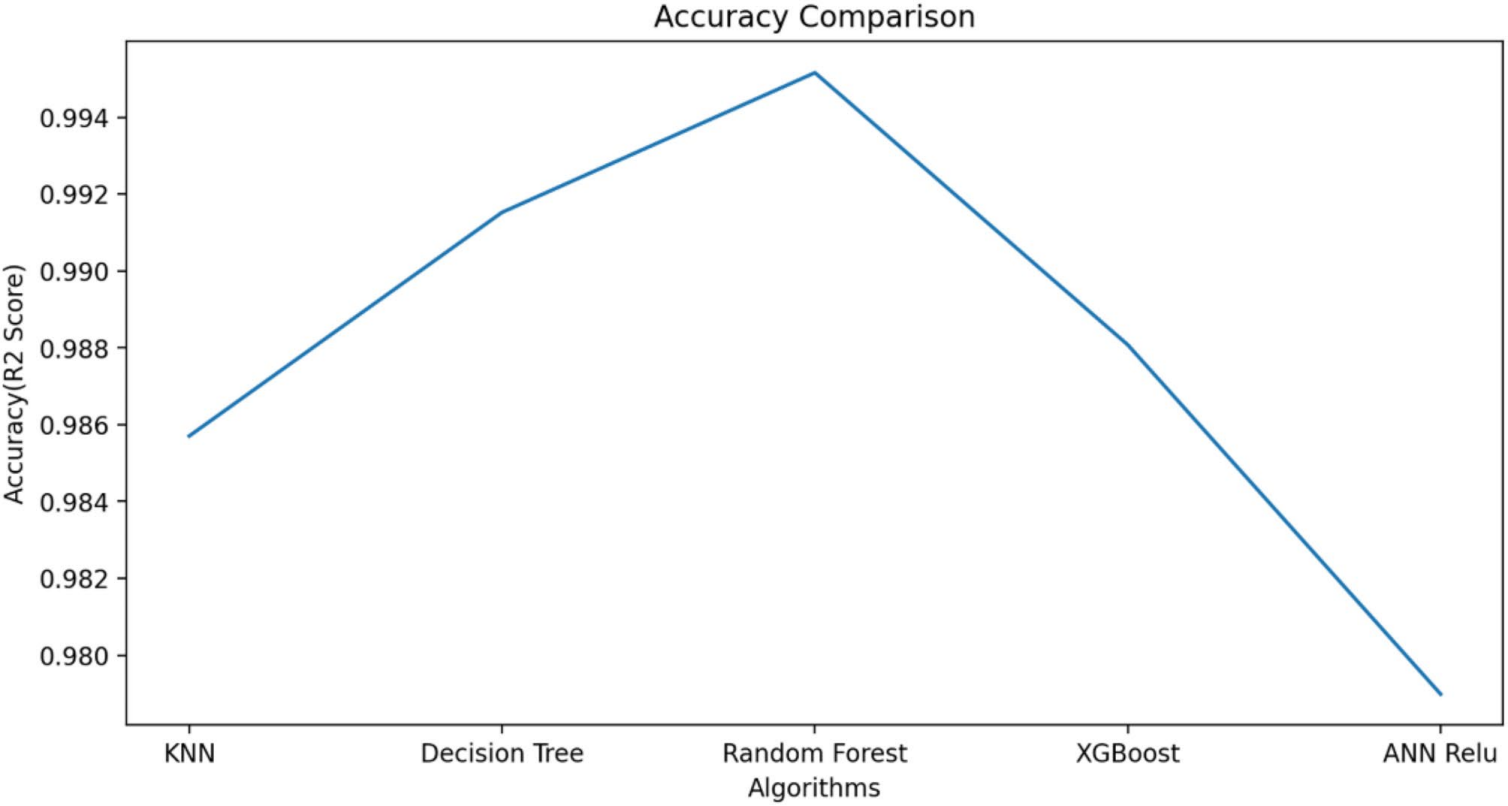
Accuracy of the ML algorithms.

**Fig. 24:**
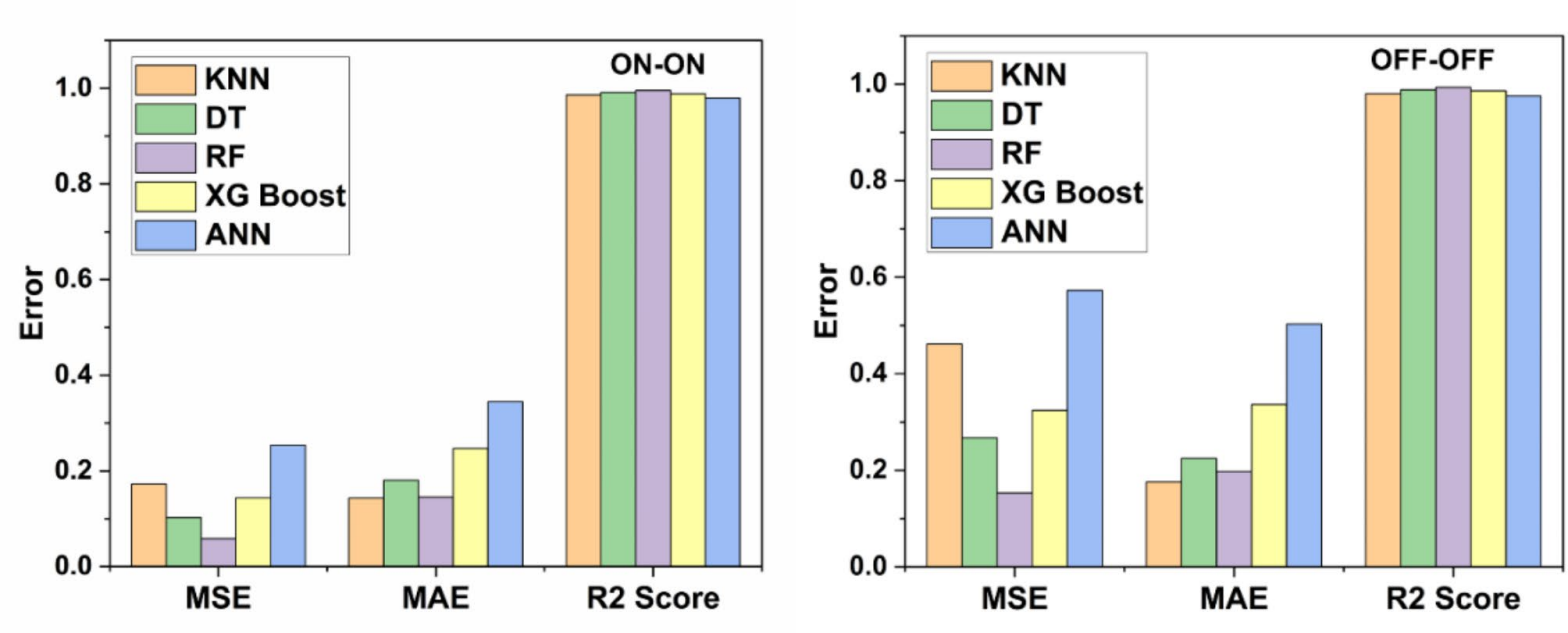
Error analysis in all configurations.

**Fig. 25:**
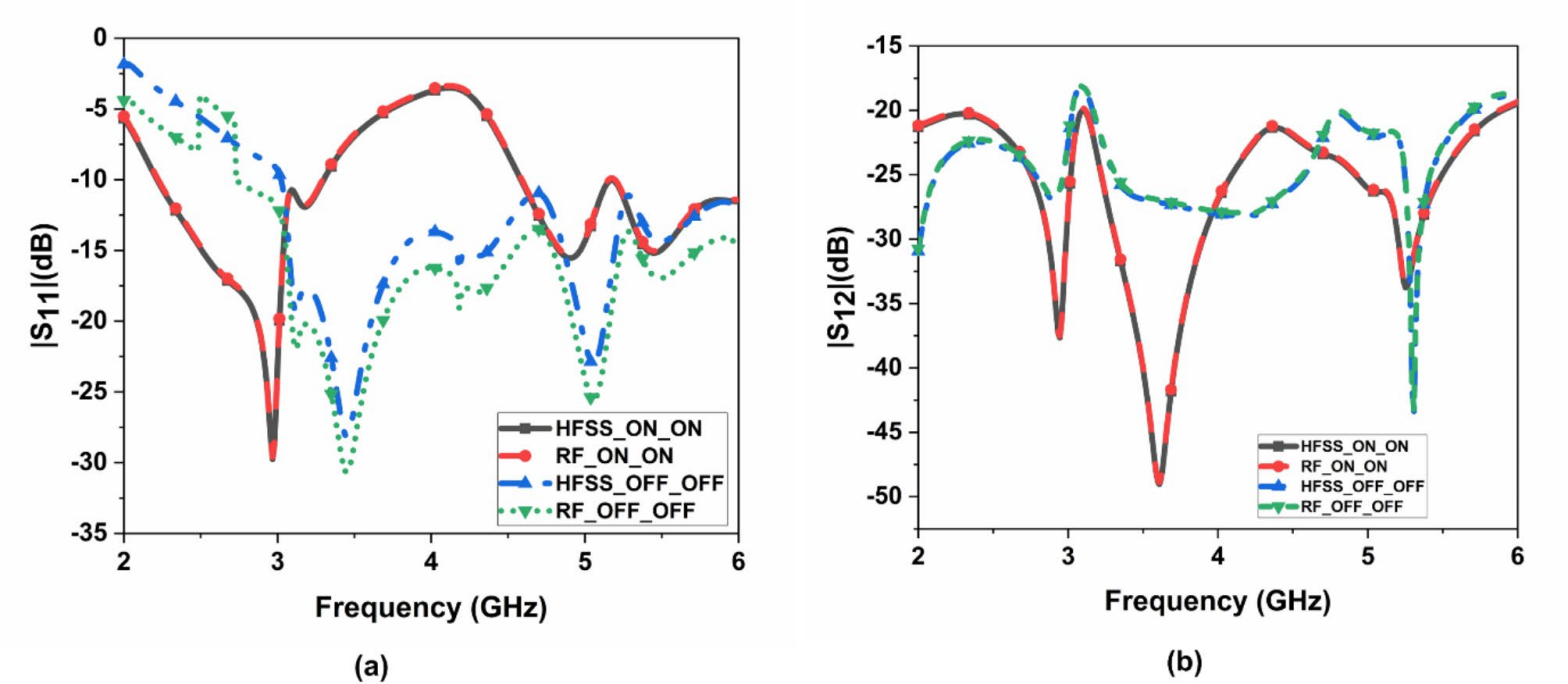
Comparison between HFSS with RF ML algorithm.

ML algorithm is used to predict the S-parameters of the suggested antenna. Choosing the ML technique will reduce the number of simulations, which will save a lot of time and error rates^20–23^. The performance of the proposed antenna depends on the length, width, and height of the rectangular DRA and the feed width of the suggested antenna. The block diagram of the ML algorithms in the suggested antenna is illustrated in Fig. 18^15,24–28^. The input and output parameters of the ML model are illustrated in Fig. 19. The KNN, RF, DT, XG, and ANN ML algorithms are applied to the suggested antenna dataset. The dataset is generated through the HFSS (High Frequency Structure Simulator version 24) software by varying the input parameters. A total of 451,527 datasets are generated in various configurations. The dataset is distributed into training and testing. 80% of the dataset is used for training the model, and 20% of the dataset is used for testing purposes to achieve better results. In various configurations, the Mean Square Error (MSE), Mean Absolute Error (MAE), and R^2^ Score are given in Tables 5 and 6, and 7. The actual vs. predicted values of various ML algorithms are illustrated in Fig. 20. The ML algorithms testing time, training time, and accuracy are illustrated in Figs. 21 and 22, and 23. The error analysis of all configurations is given in Fig. 24. From Figs. 20 and 23, and Table 7, it is clear that RF ML algorithms achieved the highest accuracy compared to others ML algorithms. The comparison of RF ML algorithm with HFSS is illustrated in Fig. 25.

**Table 5:** MSE of different configurations with ML algorithms.

Machine Learning /Configurations	KNN	DT	RF	XGBoost	ANN
ON-ON	0.1724	0.1021	0.0583	0.1437	0.2534
OFF-OFF	0.4614	0.2675	0.1532	0.3242	0.5727

**Table 6:** MAE of different configurations with ML algorithms.

Machine Learning /Configurations	KNN	DT	RF	XGBoost	ANN
ON-ON	0.1430	0.1805	0.1450	0.2466	0.3444
OFF-OFF	0.1758	0.2246	0.1980	0.3365	0.5029

**Table 7:** R^2^ score of different configurations with ML algorithms.

Machine Learning /Configurations	KNN	DT	RF	XGBoost	ANN
ON-ON	0.9857	0.9915	0.9951	0.9880	0.9789
OFF-OFF	0.98	0.9884	0.9933	0.9860	0.9752

## Conclusion

This work presents an Al_2_O_3_ DR-based FR MIMO antenna with ML-enabled through a PIN diode for 5G NR applications. The dual-band and wideband characteristics are achieved in the ON-ON and OFF-OFF configurations. More than 20 dB isolations are achieved in both configurations. MIMO parameters are determined and found within the suitable range. The simulated and measured results are in agreement. RF ML algorithms achieved highest accuracy and it is correctly predicting the S -parameters of the suggested antenna. Hence, the suggested antenna is suitable for 5G NR applications.

## Data Availability

The datasets used and/or analyzed during the current study available from the corresponding author on reasonable request.

## References

[1] Petosa, A. An overview of tuning techniques for frequency-agile antennas. *IEEE Antennas Propag. Mag.* **54** (5), 271–296 (2012).

[2] Rai, J. K., Ranjan, P. & Chowdhury, R. Recent development in reconfigurable dielectric resonator antenna and microwave filter: design and application. *Int. J. Commun Syst* **37**(14), e5859.

[3] Rai, J. K. et al. High-Gain Triple‐Band T‐Shaped dielectric resonator based hybrid Two‐Element MIMO antenna for 5G new radio, Wi‐Fi 6, V2X, and C‐Band applications with a machine learning approach. *Int. J. Commun Syst* **38**(5), e6038. (2024).

[4] Ahmad Khan, A. et al. Dual-band Mimo rectangular dielectric resonator antenna with high Port isolation for LTE applications. *Microw. Opt. Technol. Lett.* **59** (1), 44–49 (2017). 10.3390/s17010148 PMC5298721 28098807

[5] Sharma, A., Das, G. & Ravi Kumar, G. Design and analysis of tri-band dual‐port dielectric resonator-based hybrid antenna for WLAN/WiMAX applications. *IET Microwaves Antennas Propag.* **12** (6), 986–992 (2018).

[6] Rai, J. K., Ranjan, P., Chowdhury, R. & Jamaluddin, M. H. *Quad Port MIMO Al 2 O3 Ceramic Based Integrated Reconfigurable Wideband Sensing and Communication Dielectric Antenna for 5G Cognitive Radio* (IEEE Transactions on Components, Packaging and Manufacturing Technology, 2024).

[7] Khan, A. A. et al. Design of a dual-band MIMO dielectric resonator antenna with pattern diversity for wimax and WLAN applications. *Progress Electromagnet. Res. M*. **50**, 65–73 (2016).

[8] Li, K., Shi, Y. & Chang-Hong Liang Quad‐element multi‐band antenna array in the smart mobile phone for LTE MIMO operations. *Microw. Opt. Technol. Lett.* **58** (11), 2619–2626 (2016).

[9] Rai, J. K., Ranjan, P. & Chowdhury, R. Dual-band high tuning range frequency reconfigurable cylindrical dielectric resonator antenna for n7, n30, n38, n40, n41, n46, n47, n53 and n79 5G new radio application with machine learning approach. *Arab. J. Sci. Eng.*, 1–11. (2024).

[10] Malhat, H. A., Saber, H. & Zainud-Deen Low‐profile quad‐band perforated rectangular dielectric resonator antenna for wireless communications. The Journal of Engineering 8 (2017): 448–451. (2017).

[11] Anuar, S. U. et al. Triple band MIMO dielectric resonator antenna for LTE applications. *AEU-international J. Electron. Commun.* **118**, 153172 (2020).

[12] Tyagi, K., Dwivedi, A. K., Singh, S. K., Ranjan, P. & Sharma A A Four Port Dielectric Resonator Based MIMO Antenna Design for Cognitive Radio Applications IEEE Transactions on Circuits and Systems II: Express Briefs, (2022).

[13] Kajfez, D., Glisson, A. W. & James, J. Computed modal field distributions for isolated dielectric resonators. *IEEE Trans. Microwave Theory Tech.* **32** (12), 1609–1616 (1984).

[14] Das, G., Sharma, A. & Ravi Kumar, G. Dielectric resonator-based two‐element MIMO antenna system with dual band characteristics. *IET Microwaves Antennas Propag.* **12** (5), 734–741 (2018).

[15] Iqbal, A., Waly, M. I., Smida, A. & Mallat, N. K. Dielectric resonator antenna with reconfigurable polarization States. *IET Microwaves Antennas Propag.* **15** (7), 683–690 (2021).

[16] Kumar, S., Kim, K. W., Choi, H. C., Saxena, S., Tiwari, R., Khandelwal, M. K., …Kanaujia, B. K. (2018). A low profile circularly polarized UWB antenna with integrated GSM band for wireless communication. AEU-International Journal of Electronics and Communications, 93, 224–232.

[17] Kannappan, L., Palaniswamy, S. K., Kanagasabai, M., Kumar, P., Alsath, M. G. N.,Kumar, S., … Pakkathillam, J. K. (2022). 3-D twelve-port multi-service diversity antenna for automotive communications. Scientific reports, 12(1), 403. 10.1038/s41598-021-04318-0 PMC8748694 35013498

[18] Govindan, T. et al. Design and analysis of a flexible smart apparel MIMO antenna for bio-healthcare applications. Micromachines, 13(11), 1919. (2022). 10.3390/mi13111919 PMC9696043 36363940

[19] Kannappan, L. et al. Quad-port multiservice diversity antenna for automotive applications. *Sensors* **21** (24), 8238 (2021). 34960331 10.3390/s21248238 PMC8707006

[20] Goudos, S. K. et al. Design of antennas through artificial intelligence: state of the Art and challenges. *IEEE Commun. Mag.* **60** (12), 96–102 (2022).

[21] Rai, J. K., Yadav, S., Ranjan, P., Chowdhury, R. & Das, G. A compact quad element MIMO CPW fed ultra-wideband antenna for future wireless communication using machine learning optimization. *Int. J. Commun Syst* **38**(3), e5995. (2024).

[22] Sharma, Y., Zhang, H. H. & Xin, H. Machine learning techniques for optimizing design of double T-Shaped monopole antenna. *IEEE Trans. Antennas Propag.* **68** (7), 5658–5663. 10.1109/TAP.2020.296605 (July 2020).

[23] Shi, D., Lian, C., Cui, K., Chen, Y. & Liu, X. An intelligent antenna synthesis method based on machine learning. *IEEE Trans. Antennas Propag.* **70** (7), 4965–4976. 10.1109/TAP.2022.3182693 (July 2022).

[24] Cao, L. A new age of AI: features and futures. *IEEE. Intell. Syst.* **37** (1), 25–37 (2022).

[25] Mukherjee, B., Patel, P. & Mukherjee, J. A review of the recent advances in dielectric resonator antennas. *J. Electromagn. Waves Appl.* **34** (9), 1095–1158 (2020).

[26] Uddin, M. N., Islam, M. K., Ortiz, M. & Alwan, E. A. Intelligent design prediction of a circular polarized antenna for CubeSat application using machine learning algorithms. *Electronics* **12** (20), 4195 (2023).

[27] Yahya, M. S. et al. *Machine learning-optimized Compact Frequency Reconfigurable Antenna with RSSI Enhancement for long-range Applications* (IEEE Access, 2024).

[28] Rai, J. K., Choudhary, D. K., Ranjan, P. & Chowdhury, R. A compact ultrawide bandpass filter along Notch characteristics with rectangular resonator through a machine learning approach. *Int. J. Microw. Wirel. Technol.* **16**(8), 1–9. (2025).

